# Marker-based 3D motion analysis of the fingers – a systematic review

**DOI:** 10.3389/fbioe.2026.1757710

**Published:** 2026-10-30

**Authors:** Victoria Eckenweber, Mirjam Thielen, Cosima Prahm, Ursula Trinler

**Affiliations:** 1 BG Klinik Ludwigshafen, Center for Motion Analysis, Ludwigshafen, Germany; 2 Medical Faculty Heidelberg, Heidelberg University, Heidelberg, Germany; 3 Klinikum St. Elisabeth Straubing, Department of Orthopedic, Trauma and Hand Surgery, Straubing, Germany; 4 BG Klinik Tübingen, Department of Hand, Plastic, Reconstructive and Burn Surgery, University of Tuebingen, Tübingen, Germany; 5 BG Klinikum Unfallkrankenhaus Berlin, Department of Hand, Replantation, and Microsurgery and Chair of Hand, Replantation, and Microsurgery at the Charité University Medicine Berlin, Berlin, Germany

**Keywords:** biomechanics, fingers, kinematics, marker models, motion analysis, systematic review

## Abstract

**Objectives:**

No standardized marker model currently exists for recording 3D finger movements, limiting comparability across laboratories. This systematic review aims to provide a comprehensive overview of existing finger and hand models while assessing their suitability for clinical applications.

**Material and Methods:**

A systematic literature search was conducted in the PubMed and Cochrane databases. In two steps, predefined exclusion criteria were applied to titles, abstracts and full texts. The reproducibility of the studies was systematically assessed and information about methodology, validity and reliability of the models were extracted and discussed.

**Results:**

The search yielded 2,506 scientific articles, of which 38 were included in the analysis. Amongst these, 14 included the whole hand, while 14 and 10 focused solely on the thumb or the long fingers, respectively. The subjects included were mainly healthy volunteers especially in full hand models, whereas patients with osteoarthritis, carpal tunnel syndrome or rheumatoid arthritis were also involved. The movements performed ranged from directed motions to functional tasks like grasping. Markers were applied as clusters or individually. The two main kinematic approaches were vector analysis or direct kinematics.

**Conclusion:**

This review identified four hand kinematic models that achieved maximum reproducibility scores: one full-hand, two thumb, and one long finger model. However, only one of these was tested on patients, highlighting a lack of clinical verification. Additionally, suitable measures for ensuring validity and reliability are frequently missing or incomplete. Therefore, further research is needed to validate existing models and test for reliability under clinical conditions.

## 1 Introduction

Proper finger function is fundamental to the performance of activities of daily living (Lee and Jung, 2015; Goislard de Monsabert et al., 2020). Impaired finger mobility can lead to a decline in functionality and overall quality of life (Goislard De Monsabert et al., 2018; Reissner et al., 2019a). It is therefore important to have as much knowledge as possible about the function of the hand and its finger joints for clinical decision-making. The assessment of joint angles, postures and function is significant for diagnostics, treatment planning, as well as the follow up of the treatment (Cerveri et al., 2007; Reissner et al., 2019b).

Marker-based three-dimensional (3D) motion analysis is a clinical diagnostic tool, which has already established itself in the analysis of the lower extremity and is increasingly being used for the upper extremity as well. The advantages of this method include the recording of joint angles during dynamic movements, whereas other established clinical methods, such as goniometric measurements or imaging methods like MRI, are either less accurate in capturing 3D joint motion or cannot be applied during movement (Chang et al., 2013; Reissner et al., 2019b). However, the marker-based movement analysis of the finger joints is still challenging, because of the large number of segments in a limited space and its complex functional capability (Goislard de Monsabert et al., 2014). Also, while standard models exist in clinical settings for the lower limb and, to a lesser extent, for the upper limb including the wrist (Rettig et al., 2009; Jaspers et al., 2011; Nagaraja et al., 2023), there is a lack of such models for the finger joints.

Reissner et al. (2019a) previously conducted a systematic review on marker-based hand models to analyze finger movements, though focusing solely on studies involving activities of daily living of healthy participants. Other movements and cohort groups, however, have been excluded, narrowing the findings of the review. Thus, the lack of studies involving patient populations leaves an important gap regarding the clinical applicability and adaptability of marker-based hand motion models across pathological conditions. Reissner et al. (2019a) concluded that motion capture using markers are suitable for assessing the kinematics of the hand and finger joints; however, there is still limited knowledge for activities of daily living, while no preferred model for each application case could be found.

This systematic review, therefore, aims to provide a comprehensive overview of existing models, which analyze finger joint movements using marker-based 3D motion analysis. Rather than seeking a universally applicable model, a secondary objective was to assess whether any existing models demonstrate sufficient methodological clarity, completeness, and flexibility to support a broad range of clinical and research applications. Such models could serve as adaptable frameworks, balancing accuracy, feasibility, and standardization across different user groups and pathologies. The initial literature screening revealed that many studies focus exclusively on either the entire thumb or individual fingers. Since these partial models may complement each other or be integrated into broader hand models, studies that present complete kinematic models of the thumb or the long fingers were also included.

## 2 Materials and methods

The systematic review is registered in the international database PROSPERO (CRD42023379684). As we did not expect many studies to include statistical reliability and validity measures to be able to conduct a Meta-Analysis of the data, we aimed for a Synthesis Without a Meta-Analysis (SWiM) (Campbell et al., 2020).

### 2.1 Search strategy

A systematic literature search was performed in the PubMed and Cochrane databases. The search strategy included the following terms, covering keywords regarding anatomy, movement analysis and 3D-marker-based analysis (Table 1).

**TABLE 1:** Search strategy, divided into three main groups (AND) and their corresponding keywords (OR).

And	Anatomical keywords	Movement analysis	3D-marker-based
OR	Hand Finger Thumb Carpometacarpal Metacarpophalangeal interphalangeal	Movement analysis Movement analyses Motion analysis Motion analyses Motion capture Range of motion ROM Angles Kinematic	Optoelectronic Opto-electronic sensorbased Sensor-based Marker 3d Three-dimensional Three-d 3-Dimensional Model

### 2.2 Search selection and criteria

All articles were, firstly, screened by title and abstract (VE) and, subsequently, by full text (three independent reviewers VE, MT, UT). Eligibility requirements were defined based on the Participants, Intervention, Comparisons, and Outcomes (PICO) method. Participants had to be human subjects (healthy or patients), while their finger or full hand movement had to be analyzed using a marker-based model and 3D movement analysis. The outcome had to be joint angles of the included finger joints.

To be eligible for final inclusion, finger models had to represent a minimum number of physiological degrees of freedom (DoFs) for relevant joints. This requirement was crucial to ensure that finger movements could be tracked in a clinically meaningful way, reflecting the full range of joint kinematics observed in practice. For the long fingers, the metacarpophalangeal (MCP) joint must have at least two DoF and the proximal interphalangeal (PIP) and distal interphalangeal (DIP) joints at least one DoF. For the thumb, the carpometacarpal (CMC) joint had to be represented in the hand model with at least two DoF, the thumb MCP joint and the thumb interphalangeal (IP) joint with at least one DoF. All joints examined had to cover the defined DoF, however, the thumb and fingers were assessed separately. If a joint within a given ray (thumb or finger) did not meet the minimum DoF requirements, the entire ray was excluded from analysis. However, the remaining rays (e.g., the thumb despite exclusion of a finger, or *vice versa*) were still considered, provided they met the defined criteria. Figure 1 shows a flowchart presenting the full inclusion/exclusion process.

**FIGURE 1:**
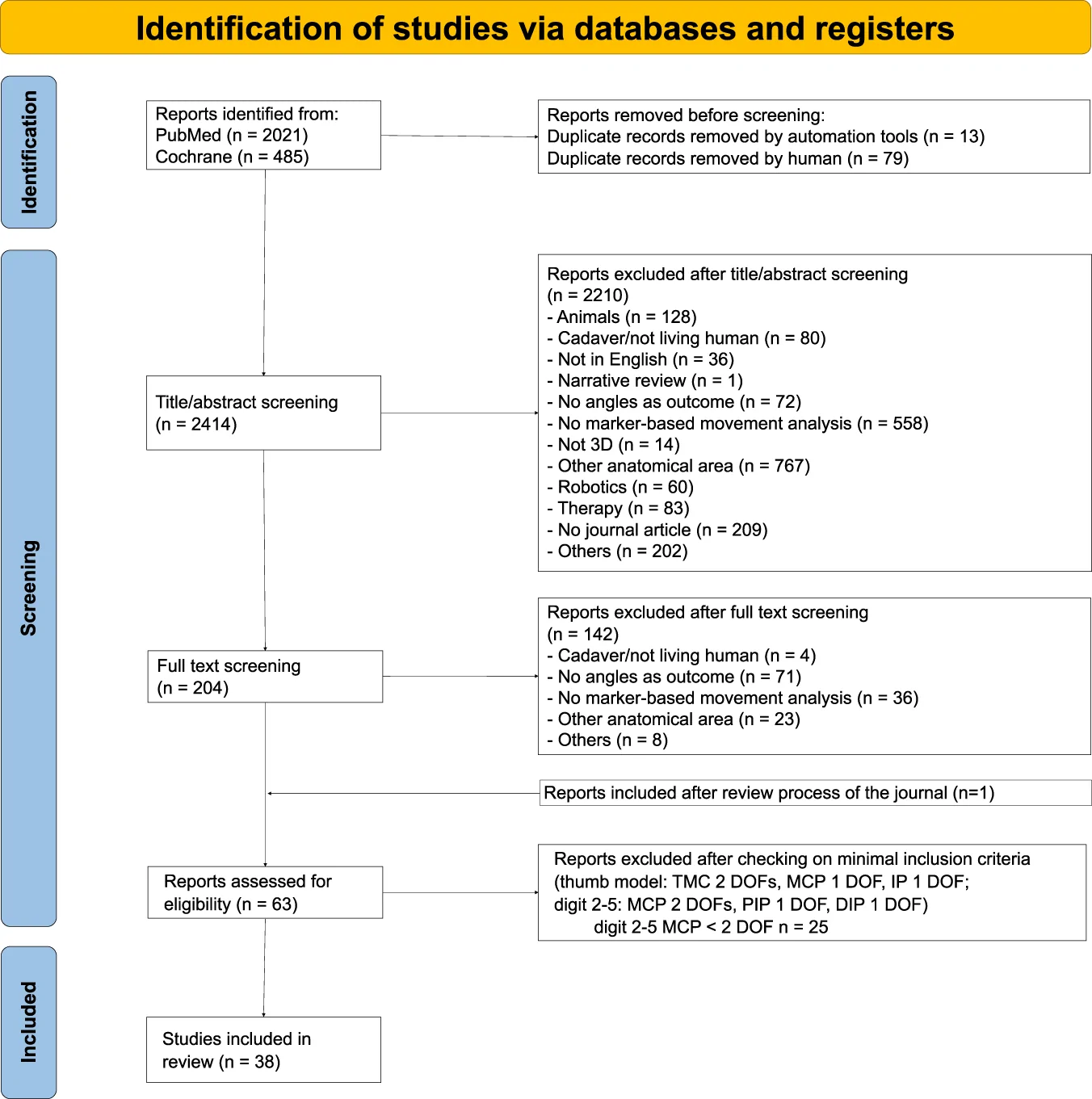
Flowchart of the inclusion and exclusion process of identified studies.

In the following step, an evaluation of the methodological description of the included studies was carried out by all authors, to rate the reproducibility of the protocol. In the event of discrepancies, the respective points were discussed within the team. The evaluation criteria were selected based on their relevance for assessing the methodological transparency and reproducibility of hand model studies. Items related to the camera system, equipment, and data processing were included to ensure that the experimental setup could be clearly understood and reproduced. Participant characteristics were considered essential for determining the populations in which the models have been tested and validated. In addition, the description of movement execution was included to allow replication of the experimental procedures. Finally, model description and statistical analysis were assessed to enable proper interpretation and contextualization of the results. Overall, these criteria were chosen to capture key aspects of methodological quality, reproducibility, and interpretability.

Each criteria could receive a maximum of one point except the description of the marker model as well as chosen statistics, which resulted in a maximum score of 10. Detailed information can be found in Table 2.

**TABLE 2:** Evaluation.

First author	Cameras	Measuring system		Subjects	Performed movements	Data processing	Model description	Statistics	Total
​	*Not stated (0P)* *Number stated (1P)*	*Not stated (0P)* *Measuring system stated (1P)*	*Not stated (0P)* *Frequency stated (1P)*	*Not stated (0P)* *1–4 Subjects (0,5P)* *≥ 5 Subjects (1P)*	*Not stated (0P)* *Inadequately described (0,5P)* *Adequately described (1P)*	*Not stated (0P)* *Data filtering mentioned (1P)*	*Not stated (0P)* *Described (1P)* *Described detailed (2P)*	*Not described (0P)* *Incompletely described (1P)* *Described (2P)*	​
Full hand models									
Baker et al., 2007	1	1	1	1	0,5	1	2	2	9,5
Cerveri et al., 2007	1	1	1	0,5	1	0	2	1	7,5
Cocchiarella et al., 2016	1	1	1	1	1	0	2	2	9
Cook et al., 2007	1	1	1	1	1	0	2	1	8
Cordella et al., 2017	1	1	1	0,5	1	0	2	1	7,5
Coupier et al., 2016	1	1	1	1	1	1	2	1	9
Goislard de Monsabert et al., 2014	1	1	1	0,5	1	0	2	1	7,5
Lee and Jung, 2016	1	1	1	1	1	1	2	2	10
Leitkam et al., 2014	1	1	1	1	1	0	2	0	7
Leitkam and Reid Bush, 2015	1	1	0	1	1	0	2	2	8
Ma’touq et al., 2018	0	1	0	1	1	0	2	0	5
Metcalf et al., 2008	1	1	1	1	1	1	2	1	9
Nataraj and Li, 2013	0	1	1	1	1	0	2	1	7
Sancho-Bru et al., 2014	1	1	0	1	0,5	0	1	1	5,5
Thumb models									
Carpinella et al., 2006	1	1	1	1	1	1	1	1	10
Cerveri et al., 2008	1	1	1	1	1	0	2	2	9
Chang and Pollard, 2008	0	1	0	1	0,5	0	2	1	5,5
Coppola et al., 2018	0	1	0	1	0,5	1	1	2	6,5
Curran et al., 2019	1	1	1	1	1	0	2	2	9
Gustafsson et al., 2018	1	1	1	1	1	1	2	2	10
Hamann et al., 2014a	1	1	1	1	1	0	1	2	8
Hamann et al., 2014b	1	1	1	1	1	0	1	2	8
Kodama et al., 2021	1	1	1	1	1	0	1	1	7
Li and Tang, 2007	0	1	1	1	1	0	1	2	7
Lin et al., 2011	0	1	1	1	1	0	2	2	8
Marquardt et al., 2014	0	1	1	1	1	1	1	2	8
Tang et al., 2008	0	1	1	1	0,5	0	2	1	6,5
Zhang et al., 2005	0	1	1	1	1	0	2	1	7
Long finger models									
Degeorges et al., 2005	1	1	1	1	0,5	0	2	0	6,5
Gharib et al., 2015	1	1	1	1	1	0	0	2	7
Hensler et al., 2020	0	1	0	1	1	0	1	2	6
Lee and Jung, 2015	1	1	1	1	1	1	2	2	10
Lester et al., 2012	1	1	1	1	1	1	1	1	8
Metcalf and Notley, 2011	1	1	1	1	1	1	2	0	8
Metcalf et al., 2020	1	1	1	1	1	0	2	1	8
Speirs et al., 2001	0	1	0	1	0	0	2	1	5
Wang et al., 2013	1	1	1	1	0,5	1	2	2	9,5
Warlow and Lawson, 2012	1	1	1	1	1	0	2	2	9

### 2.3 Data extraction and analysis

Data was extracted independently by two reviewers (VE, UT), while disagreements were discussed with the third reviewer (MT). All extracted data was compiled in a tabular overview. As we did not find many studies which included a statistical reliability and validity analysis of their hand model, we were not able to do a Meta-Analysis. The reported reliability and validity measures were summarized descriptively. Furthermore, the included studies were evaluated regarding their application to established methodological guidelines, such as the International Society of Biomechanics recommendations (ISB) for hand model parameters (e.g., segment and coordination system definition, degree of freedoms) (Wu et al., 2005).

## 3 Results

The search yielded 2,506 results. After full-text screening, 63 studies were included. Of these 63 studies, 38 met the aforementioned requirements for the model (Figure 1). 14 of these studies included a full hand model (i.e., all long fingers and thumb), whereas 14 and 10 only presented models of the thumb or single long fingers, respectively. The evaluation of the studies resulted in total scores between 5 and 10 points, with the detailed evaluation shown in Table 2.

### 3.1 Participants

Of the 38 included studies, the number of subjects varied from one (Goislard de Monsabert et al., 2014; Cordella et al., 2017) to 40 (Lester et al., 2012; Gharib et al., 2015) with an average of 17 participants per study (Table 3). In total, 28 studies only involved healthy subjects, while six studies involved both patients and healthy subjects (Carpinella et al., 2006; Lester et al., 2012; Hamann et al., 2014a; Marquardt et al., 2014; Leitkam and Reid Bush, 2015; Hensler et al., 2020) and four studies exclusively focused on patients (Metcalf and Notley, 2011; Hamann et al., 2014b; Gharib et al., 2015; Kodama et al., 2021) (Figure 2, left). Studies of the whole hand focused mainly on healthy participants, except Leitkam and Reid Bush (2015), who compared 22 healthy participants with 21 patients with arthritis. The patient cohort of partial hand models manifested various conditions, including osteoarthritis of the CMC joint of the thumb (Hamann et al., 2014a; Hamann et al., 2014b), osteoarthritis of the PIP joint (Hensler et al., 2020), carpal tunnel syndrome (Marquardt et al., 2014; Kodama et al., 2021), stroke (Carpinella et al., 2006; Gharib et al., 2015), rheumatoid arthritis of the MCP joints (Lester et al., 2012), or general impairments resulting from finger injuries (Metcalf and Notley, 2011). Data collection in all included studies was limited to a single assessment time point. No study reported longitudinal follow-up data or repeated measurements. Details of the included participants are shown in Table 3.

**TABLE 3:** Subjects, performed movements, measured joints, validation and reliability.

First author	Participants	Performed movements	Measured joints	Validation and reliability
Full hand models				
Baker et al., 2007	20 healthy (17 women, 3 men)	Typing on a keyboard, three 1-min trials at 15-min, 20-min and 24-min	Digit 2–5: MCP, PIP Thumb: MCP, IP	--
Cerveri et al., 2007	4 „normal” subjects	(1) Voluntary flexion-extension of the thumb, (2) voluntary abduction–adduction of the thumb, (3) tip-to-tip grasping of index and thumb and (4) cylinder grasping	Digit 2–5: MCP, PIP, DIP Thumb: CMC, MCP, IP	Reliability Intra-subject repeatability
Cocchiarella et al., 2016	8 healthy (4 women, 4 men)	Static motions: Gripping a cylindric tube, gripping a cap	Digit 2–5: MCP Thumb: CMC, MCP, IP	--
Cook et al., 2007	5 healthy (2 women, 3 men)	Postures tested: 30°, 60°, and 90° of finger MCP and PIP flexion (all except thumb); 20° of radial deviation; 20° of ulnar deviation; 30° and 60° of wrist extension; and 30° and 60° of wrist flexion. The finger flexion angles of 30°, 60°, and 90° were obtained by placing the hands on the appropriate triangular blocks of 150°, 120° and 90°, respectively	Digit 2–5: MCP, PIP Thumb: CCP, IP	Validation Compared to goniometer measurement, linear regressions of groups of flexion/extension data
Cordella et al., 2017	1 healthy	Different grasping actions with tripod, pinch and palmar grasp; hand starting with four fingers fully extended and thumb adducted	Digit 2–5: MCP, PIP, DIP Thumb: CMC, MCP, IP	Validation Model applied to robotic hand Reliability Application of markers 5 times, results only for the TMb marker
Coupier et al., 2016	20 healthy (10 women, 10 men) (both hands)	Flexion/extension of long fingers, abduction/adduction of long fingers, flexion/extension of the thumb, abduction/adduction of the thumb	Digit 2–5: MCP, PIP, DIP Thumb: TBJ (thumb basis joint→radio-scaphoid, scapho-trapezial and CMC joint), MCP, IP	Validation Compared with data measured with a goniometer by a trained clinician on the same participants Reliability - Reproducibility testing was performed on three cadaver hands, following a methodology that had been published earlier (Coupier et al., 2014) - Inter-rater and intra-rater: Two operators affixed the markerset on three occasions
Goislard de Monsabert et al., 2014	1 healthy	Flexion motions of long fingers, grasping a 7 cm diameter field hockey ball	Digit 2–5: MCP, PIP, DIP Thumb: CMC, MCP, IP	--
Lee and Jung, 2016	10 healthy students	Pick up an object, lift it up and maintain posture for 3 s (four cylinders and four square pillars)	Digit 2–5: MCP, PIP, DIP Thumb: CMC, MCP, IP	--
Leitkam et al., 2014	9 healthy (5 women, 4 men)	Maximum MCP flexion, maximum DIP and PIP flexion, maximum finger extension, maximum finger spread between each of the neighboring digits two through five, minimum and maximum thumb inclination angle, and minimum and maximum thumb azimuth angle	Digit 2–5: MCP, PIP, DIP Thumb: CMC, MCP, IP	Validation Theoretical hand model is compared against experimental data
Leitkam and Reid Bush, 2015	22 healthy (10 women, 10 men) 21 with arthritis (16 women, 5 men)	Full flexion of the finger MCP joints, full flexion at the PIP and DIP joints with the MCP joints extended, fingers spread with a flat hand, rotation of the thumb, rotation of the thumb around the hand with the thumb held as tight to the palm as possible, full flexion of the thumb MCP and IP joints, full extension of all joints of the hand	Digit 2–5: MCP, PIP, DIP Thumb: CMC, MCP, IP	--
Ma’touq et al., 2018	9 healthy (2 women, 7 men)	Grasp and release a cylinder, grasping a small paper clip (thumb and pinky opposition)	Digit 2–5: MCP, PIP, DIP Thumb: CMC, MCP, IP	Validation Consistency and accuracy between estimated and measured data Reliability: -
Metcalf et al., 2008	Static: 8 healthy, dynamic: 10 healthy	Static positions, grasping and lifting a sphere	Digit 2–5: MCP, PIP, DIP Thumb: CMC, MCP, IP	Validation Only for wrist Reliability -Inter-rater-reliability
Nataraj and Li, 2013	6 healthy (1 woman, 5 men)	Thumb circumduction, index finger flexion/extension; pinching	Digit 2: MCP, PIP, DIP Thumb: CMC, MCP, IP	--
Sancho-Bru et al., 2014	5 healthy (2 women, 3 men)	Grasping and raising 3 different objects (a cone, a sphere and a cylindrical object)	Digit 2–5: MCP, PIP, DIP Digit 4 & 5: CMC Thumb: CMC, MCP, IP	Validation Comparison with use of goniometers (only for subject 5, without DIP joints and abduction/adduction of the wrist); paired T-test p > 0,05 Reliability - Repeatability testing: Subjects repeat the tasks 3 times - Reproducibility testing by 3 different operators measuring static hand posture for subject 2 and 5 for grasping the cone
Thumb models				
Carpinella et al., 2006	14 healthy 2 after stroke (1 woman, 1 man)	Static positions (hand closed, hand grasping a cylinder d = 40mm, hand grasping a cylinder d = 80mm, hand opened, hand openend thumb flexed and adducted)	Thumb: MCP, IP (Digit 2–5: MCP only 1 DoF, PIP)	Reliability Variance within-subject
Cerveri et al., 2008	10 healthy (4 women, 6 men)	Circumduction movements of the thumb	Thumb: CMC	--
Chang and Pollard, 2008	24 healthy (12 women, 12 men)	Circumduction, star pattern, abduction/adduction and flexion/extension movements	Thumb: CMC	--
Coppola et al., 2018	16 healthy (8 women, 8 men)	16 thumb swiping gestures on a tablet	Thumb: CMC, MCP, IP	--
Curran et al., 2019	10 healthy (10 men)	Thumb flexion, extension, opposition, radial/palmal abduction/adduction, finger flexion/extension, total thumb ROM -All points in space where thumb-tip and each fingertip can contact each other (functional workspace) −12 activities of daily living (tip-pinch, tripod pinch, lateral pinch, cylinder grip, spherical grip)	Thumb: CMC, MCP, IP	Validation -With from wood constructed male hand -Goniometer measurement
Gustafsson et al., 2018	19 healthy (12 women, 7 men)	Texting tasks with a keypad phone and a touchscreen phone	Thumb: MCP	--
Hamann et al., 2014a	36 (18 healthy (9 women, 9 men) 18 with stage 2/3 CMC 1 osteoarthritis (all women))	Maximum active opposition and reposition of the thumb	Thumb: CMC, MCP	--
Hamann et al., 2014b	18 women with stage 2/3 CMC osteoarthritis of the thumb	Maximum active opposition and reposition of the thumb with and without wearing orthoses	Thumb CMC and MCP	--
Kodama et al., 2021	14 after carpal tunnel release (18 hands)	Circumduction of the thumb (extension, abduction, flexion and return to neutral position)	Thumb: CMC, MCP, IP	--
Li and Tang, 2007	15 healthy (women)	Opposition and circumduction of the thumb; other fingers fixed	Thumb: CMC, MCP, IP	--
Lin et al., 2011	16 healthy (7 women, 9 men)	Six common ADLs were performed from the neutral position (except for the lateral pinch and power grip): tip pinch; palm pinch; lateral pinch (twist a key); cylinder grip (diameter 6.3 cm); spherical grip (ball with diameter of 6.5 cm); and power grip (grip a hammer)	Thumb CMC, MCP, IP	--
Marquardt et al., 2014	11 healthy 11 with carpal tunnel syndrome	Opposition and circumduction of the thumb	Thumb: CMC, MCP, IP	--
Tang et al., 2008	15 healthy (women)	Circumduction and operational motion (move the thumb randomly and continuously in a self-defined comfortable motion for 60s per trail) (hand and other fingers fixated)	Thumb: CMC, MCP, IP	--
Zhang et al., 2005	28 healthy (14 women, 14 men)	Circumduction of the thumb	Thumb: CMC, MCP, IP	--
Long finger models				
Degeorges et al., 2005	6 healthy (2 women, 4 men)	Repeated flexion/extension	Digit 2–5: MCP, PIP, DIP	--
Gharib et al., 2015	40 hemiparetic stroke patients (16 women, 24 men)	Abduction (ring, middle, index and thumb), extension (MCP/PIP for index, ring and middle fingers)	Digit 2–4: MCP, PIP Thumb: CMC (only 1 DoF)	--
Hensler et al., 2020	30 (10 healthy, 10 patients with PIP joint osteoarthritis CapFlex-PIP implant, 10 patients with PIP joint osteoarthritis with swanson finger joint implant)	Lateral deviation in different PIP flexion angles	Digit 2 or 3: PIP	Validation: - Reliability Test-retest reliability was assessed by the primary author and evaluated with intraclass correlation coefficient method (ICC 0,93)
Lee and Jung, 2015	10 healthy sport students (10 men)	Gripping a cylinder	Digit 2–5: MCP, PIP, DIP	Validation Hand mock-up (3D printed hand) finger position (in °) vs. angle calculated with 1, 2, 3 markers or cluster analysed with mean absolute angle difference
Lester et al., 2012	40 (10 younger adults, 10 older healthy adults, 10 with rheumatoid arthritis treatment non-surgical, 10 with rheumatoid arthritis post-surgery)	pinch grip, a key pinch, making a fist, spreading the fingers of the hand	Digit 2–5: MCP	--
Metcalf and Notley, 2011	8 with impairment and injuries of their fingers (7 women, 1 man)	Grasping and lifting a cup	Digit 2–5: MCP, PIP, DIP	--
Metcalf et al., 2020	10 healthy (5 women, 5 men)	Flexion and extension	Digit 2–5: PIP, DIP	Validation CT-scans to check reference joint angles Reliability: -
Speirs et al., 2001	24 healthy	Neutral and 30° flexed MCP postures	Index: MCP	Reliability Test-retest reliability with 2 replicates; marker positions were reconstructed based on stereoradiographs
Wang et al., 2013	19 baseball pitchers	Baseball pitches	Digit 2 and 3: MCP, PIP, DIP	--
Warlow and Lawson, 2012	13 healthy (6 women, 7 men)	Full flexion/extension of DIP and PIP joint, full flexion/extension and abduction/adduction of MCP joint	Index: MCP, PIP, DIP	--

**FIGURE 2:**
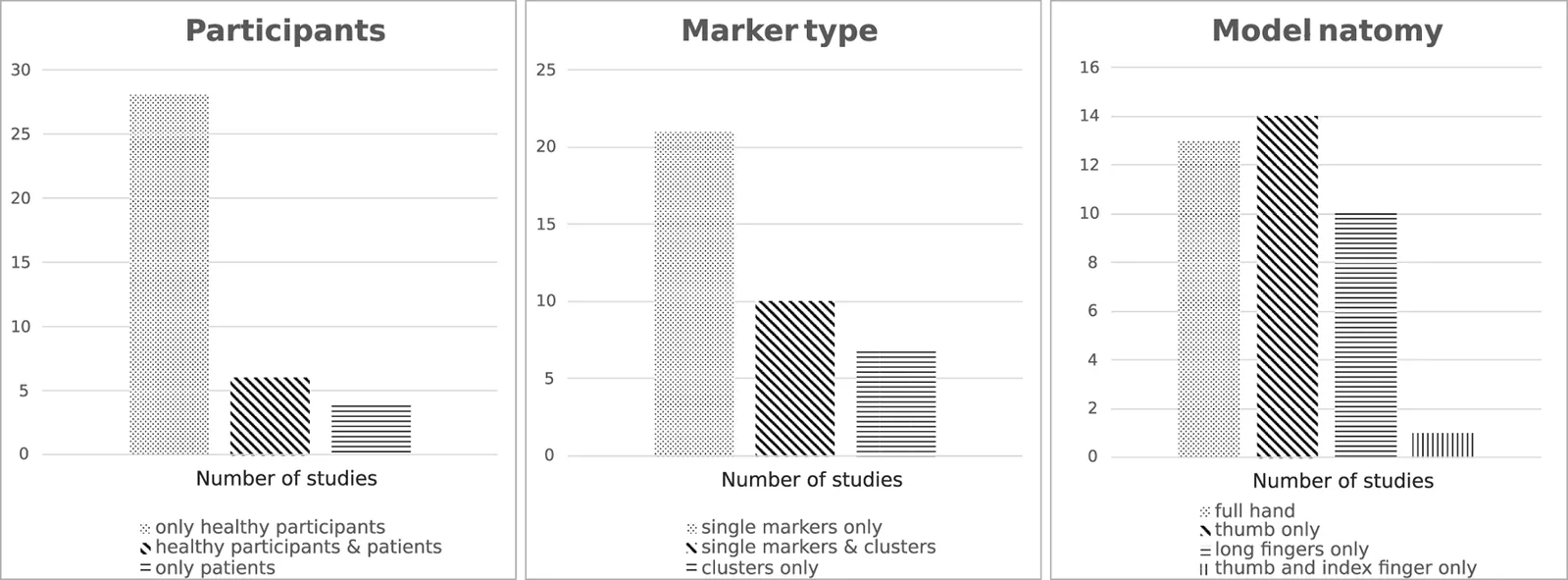
Visual overview of number of studies regarding participant characteristics (healthy participant or patient–left), marker types (single or cluster marker–middle), and model anatomy structure (right).

### 3.2 Performed movements

The movements assessed in the included studies ranged from isolated range of motion tasks to functional activities of daily living (Table 3). For the former, flexion/extension for the long fingers (nine studies) and thumb (six studies), ab-/adduction of the MCP joint in both long fingers (six studies) and thumb (1 study), maximal finger spread of the long fingers (two studies), as well as circumduction (nine studies) and opposition (four studies) for the thumb were included. These postures were not only captured dynamically but also statically (Speirs et al., 2001; Carpinella et al., 2006; Cook et al., 2007; Metcalf et al., 2008). The latter included typing on a keyboard (three studies), swiping with the thumb on a display (one study), different grasping tasks (12 studies), grasping and lifting an object (three studies), pinching in different variations (five studies or specific tasks like passive lateral deviation of the long fingers (Hensler et al., 2020), making a fist (Lester et al., 2012) or baseball throwing (Wang et al., 2013).

### 3.3 Measurement equipment

The measurement devices hailed from various brands (Table 4), including Vicon (most commonly used), BTS, Optotrack, Qualisys, Watsmart, Motion Analysis Corporation, CamBar B2 C4, and Venus 3D. The quantity of cameras ranged from three (Gharib et al., 2015) to 14 (Lee and Jung, 2015) for studies analyzing solely the long fingers, five (Zhang et al., 2005) to 18 (Gustafsson et al., 2018) for studies of the thumb, and five (Baker et al., 2007; Cook et al., 2007) to twelve (Cocchiarella et al., 2016) for full hand studies. Ten studies did not report the exact number of cameras, especially studies analyzing the thumb.

**TABLE 4:** Measurement equipment, recording frequency and data filtering.

First author	Measuring system brand	Camera quantity	Marker type	Recording frequency (In Hz)	Data filtering (type, cut-off-frequency)
Full hand models					
Baker et al., 2007	Vicon	5	Passive	60	7th order lowpass butterworth, 5 Hz
Cerveri et al., 2007	Smart by BTS	6	Passive	60 or 120	H FIR real-time low pass filters tuned with the acquisition frequency
Cocchiarella et al., 2016	MAC	12	Passive	60	No specification
Cook et al., 2007	Vicon	5	Passive	60	No specification
Cordella et al., 2017	BTS SMART-D motion capture system	7	Passive	60	No specification
Coupier et al., 2016	Vicon	8	Passive	100	Lowpass butterworth
Goislard de Monsabert et al., 2014	Optotrack	9	Active	25	No specification
Lee and Jung, 2016	Vicon	10	Passive	50	2nd order lowpass butterworth, 6 Hz
Leitkam et al., 2014	Qualisys	7	Passive	No specification	No specification
Leitkam and Reid Bush, 2015	Qualisys	7	Passive	No specification	No specification
Ma’touq et al., 2018	Vicon	No specification	Passive	No specification	No specification
Metcalf et al., 2008	Vicon	6	Passive	100	No specification
Nataraj and Li, 2013	Vicon	No specification	Passive	100	No specification
Sancho-Bru et al., 2014	Vicon	8	Passive	No specification	No specification
Thumb models					
Carpinella et al., 2006	Smart BTS	9	Passive	60	Low-pass butterworth digital filter, cut-off frequency of 6 Hz
Cerveri et al., 2008	Smart BTS	6	Passive	60	No specification
Chang and Pollard, 2008	Vicon	No specification	Passive	No specification	No specification
Coppola et al., 2018	Optotrack	No specification	Active	No specification	4th order lowpass butterworth, 10 Hz
Curran et al., 2019	Motion analysis corporation santa rosa, CA, USA	12	Passive	120	No specification
Gustafsson et al., 2018	Vicon	18	Passive	250	Woltring (no further specification)
Hamann et al., 2014a	Vicon	6	Passive	100	No specification
Hamann et al., 2014b	Vicon	6	Passive	100	No specification
Kodama et al., 2021	VENUS 3D	12	Passive	100	No specification
Li and Tang, 2007	Vicon	No specification	Passive	60	No specification
Lin et al., 2011	Motion analysis corporation	No specification	No specification	60	No specification
Marquardt et al., 2014	Vicon	No specification	Passive	100	Lowpass butterworth, 10 Hz
Tang et al., 2008	Vicon	No specification	Passive	60	No specification
Zhang et al., 2005	Vicon	5	Passive	120	No specification
Long finger models					
Degeorges et al., 2005	Vicon	6	Passive	50	No specification
Gharib et al., 2015	Qualisys	3	Passive	120	No specification
Hensler et al., 2020	CamBar B2 C4	No specification	Passive	No specification	No specification
Lee and Jung, 2015	Vicon	14	Passive	50	No specification
Lester et al., 2012	Vicon	12	Passive	60	Lowpass butterworth, 1 Hz
Metcalf and Notley, 2011	Vicon	12	Passive	100	Woltring (mean square error value = 10)
Metcalf et al., 2020	Vicon	12	Passive	100	No specification
Speirs et al., 2001	WATSMART	No specification	Passive	No specification	No specification
Wang et al., 2013	Motion analysis corporation	8	Passive	500	4th order lowpass butterworth, 6 Hz
Warlow and Lawson, 2012	Vicon	6	Passive	100	No specification

A key factor when using small markers is the resolution of the applied measurement set up. However, only three studies have included this information in their article (Degeorges et al., 2005; Metcalf and Notley, 2011; Coupier et al., 2016). Passive markers were used in all but three studies. In two studies, active markers were used (Goislard de Monsabert et al., 2014; Coppola et al., 2018) and in one study the information about the marker type could not be determined (Lin et al., 2011). Table 4 shows detailed information about the measurement equipment.

### 3.4 Recording frequency and data processing

Recording frequencies ranged between 25 Hz (Goislard de Monsabert et al., 2014) and 500 Hz (Wang et al., 2013), with 60 Hz being the most frequently adopted frequency, followed by 100 Hz (Table 4). Similar high to low measurement frequencies were present in studies including the whole hand as well as studies analyzing only one digit (thumb). Eight studies did not specify this information at all. Data filtering procedures were defined in 10 cases, where either a Butterworth lowpass or a Woltring filter were applied (Table 4).

### 3.5 Examined segments and joints

The applied models varied regarding their main anatomical structure (Figure 2, right). Thirteen studies included the full hand (i.e., thumb and digit 2–5), while one study analyzed solely the thumb and the index finger (Nataraj and Li, 2013). Ten of the included studies focused on the long fingers (digit 2–5). Here, most studies included all four digits, one included digits 2–4 (Gharib et al., 2015), one digit two and 3 (Hensler et al., 2020) and two focused only on the index finger (Speirs et al., 2001; Warlow and Lawson, 2012). The total number of segments of the models varied between two and 20, depending on the included digits and detailed representation (Table 6). While long fingers are mostly represented by two (proximal, and middle phalanx) or three segments (including distal phalanx) and the metacarpal bones are either merged into one segment (palm) or fully excluded, the thumb mostly consisted of the proximal phalanx and metacarpal bone and, sometimes, additionally the distal phalanx.

Fourteen studies covered exclusively the thumb (Table 3). In most of the studies which included the thumb, all joints (CMC, MCP and IP) were recorded, except two studies only measured the CMC and the MCP joint (Hamann et al., 2014a; Hamann et al., 2014b), two studies measured the MCP and the IP joint of the thumb (Carpinella et al., 2006; Baker et al., 2007), two studies only included the CMC (Cerveri et al., 2008; Chang and Pollard, 2008), and two studies recorded only the MCP joint (Cook et al., 2007; Gustafsson et al., 2018). Similar to the thumb, most studies which only included the long fingers considered all joints (MCP, PIP and DIP). In three studies, only the MCP joint and the PIP joint were examined (Baker et al., 2007; Cook et al., 2007; Gharib et al., 2015), three studies focused on the MCP joint (Speirs et al., 2001; Lester et al., 2012; Cocchiarella et al., 2016) and one study only examined the PIP joint (Hensler et al., 2020). One study additionally measured the CMC joints of digit four and five (Sancho-Bru et al., 2014) (Table 3).

### 3.6 Marker setup

Markers were applied to the forearm, hand and/or fingers in 21 studies using single markers on the skin, in seven studies using marker clusters, and in nine studies using a combination of both (Table 5; Figure 2). Lee and Jung (2015) compared marker sets with either single markers or clusters. Marker size ranged from 3 mm (Sancho-Bru et al., 2014; Coupier et al., 2016; Hensler et al., 2020; Metcalf et al., 2020) to 17 mm (Degeorges et al., 2005), with 4 mm being the most common, followed by a diameter of 3 mm.

**TABLE 5:** Marker setup (cluster/single/virtual, quantity, size, location).

First author	Markers	Quantity	Diameter, shape	Location
Full hand models				
Baker et al., 2007	Single markers	21	4 mm, spherical	- Distal forearm between radius and ulna - Radial and ulnar styloid −3 hand markers: Proximal 2nd and 4th metacarpal bone and distal 2nd metacarpal bone - Digit 2–5: Distal MCP joint, distal PIP joint, distal DIP joint - Thumb: Lateral aspect of MCP joint, lateral aspect of IP joint, lateral aspect of nail
Cerveri et al., 2007	Single markers	24	Hemispherical, spherical for the wrist	−4 markers rigidly linked with each other around the wrist -Digit 2–5: Markers on MCP, PIP, DIP and fingertip -Thumb: Base of metacarpal segment, proximal phalanx and distal phalanx and on the fingertip
Cocchiarella et al., 2016	Cluster Single markers Virtual markers	18 × 3 markers 42 calibration m., 54 tracking m	4 mm, hemispherical	Calibration markers - Markers on the tips of the distal phalanges - Anterior and posterior MCP, PIP, DIP joints - Thumb: tip of the distal phalanx, anterior and posterior CMC, MCP, IP and lateral aspect of the CMC joint −4 markers on forearm and ulnar/radial styloid Tracking markers - Marker triads on each finger segment - Marker triads between metacarpal bone 2–3 and 4–5 and the distal aspect of the posterior forearm Virtual markers Distal metacarpal endpoints (between anterior and posterior MCP joint markers)
Cook et al., 2007	Single markers	21	4 mm, spherical	-Distal forearm between radius and ulna -Lateral and medial wrist -hand: proximal 4th MCP, proximal 1st MCP, distal 2nd MCP -Digit 2–5: Distal MCP, on PIP joint, on DIP joint -Thumb: Medial aspect of MP, medial aspect of IP, medial aspect of nail
Cordella et al., 2017	Single markers	25	6 mm, spherical	-Thumb (4 markers): CMC, MCP, IP, fingertip -Digit 2–5: MCP, PIP, DIP, fingertip -Hand: 3 on the hand dorsum −1 marker on wrist dorsum
Coupier et al., 2016	Cluster	20 × 4 markers (on pins)	Hand: 3 mm Wrist: 10 mm Spherical	- Wrist: Dorsal wrist - hand: on metacarpal bones, proximal and middle phalanges and fingertips
Goislard de Monsabert et al., 2014	Cluster	17 × 3 markers	No specification	- One markertriad per proximal, middle and distal phalanx (not on bony landmarks)
Lee and Jung, 2016	Single markers Virtual markers	41 + 15 „floating markers“ 4	4 mm, hemispherical	- Proximal heads of MCP, IP, PIP, DIP joints - Fingertips - Proximal heads of the second and fifth carpal bones at the CMC joints −15 float markers at the mid-points of the phalanges on the thumb and four fingers - Four virtual markers were used to calculate the joints centers of the third and fourth CMC joints, wrist and forearm
Leitkam et al., 2014	Cluster	16 × 4 markers	4 mm, 6 mm, spherical	−16 marker pods with 4 markers per pod -One 4 mm marker pod on each phalanx segment -One 6 mm marker pod on the third metacarpal bone -One 6 mm marker pod on the first metacarpal bone
Leitkam and Reid Bush, 2015	Cluster	16 × 4 markers	4 mm, 6 mm, spherical	−16 marker pods with 4 markers per pod -One 4 mm marker pod on each phalanx segment -One 6 mm marker pod on the third metacarpal bone -One 6 mm marker pod on the first metacarpal bone
Ma’touq et al., 2018	Cluster Single markers	2 × 3 markers (on L-shaped plates) 19	No specification	Cluster - On first and third metacarpal bone Single markers - On head of metacarpal bones, phalanges and fingertips
Metcalf et al., 2008	Single markers Virtual marker	26 1	3 mm, hemispherical	- Distal head of ulnar and radial styloid process - Dorsal aspect of radius and ulna - Proximal head of 1st, 2nd and 5th metacarpal bone at CMC joint - Distal head of 1st to 5th metacarpal bone - Distal head of each phalanx (fingertips) - Virtual marker halfway between CMC 2 and CMC 5
Nataraj and Li, 2013	A: Cluster (tripods) B: Cluster Single markers C: Cluster	2 × 3 on pin, 1 × 3 on T-shaped plate 2 × 3 on pin, 1 × 3 on T-shaped plate 8 2 × 3 on pin, 1 × 3 on T-shaped plate, 4 × 3 on L-shaped plate	4 mm, spherical	Cluster - On pins: thumb nail and index nail - T-shaped cluster on second metacarpal bone - L-shaped cluster on first metacarpal bone, proximal phalanx of the thumb, proximal phalanx of index finger and middle phalanx of index finger Single markers - two markers on first metacarpal bone, thumb and index proximal phalanges and index middle phalanx
Sancho-Bru et al., 2014	Single markers	29	3 mm, spherical	-Longfingers: First marker on the metacarpal base, second marker on the knuckle, third on the proximal interphalangeal (PIP) joint, forth on the distal interphalangeal (DIP) joint and fifth marker on the nail -Thumb: First marker on the metacarpal base (T1), second and third markers (T2 and T3) on dorsum and lateral side of the MCP joint, forth on the IP joint (T4) and fifth marker on the nail (T5) -Wrist: Radial, ulnar styloid process, wrist dorsum and forearm with 2.5 cm distance, aligned with digit 3
Thumb models				
Carpinella et al., 2006	Single markers	17	6mm, hemispherical	−2 markers on radial/ulnar styloid -Digit 2–5: Distal head of metacarpal bone, proximal and middle phalanx -Thumb: Distal head of metacarpal bone, proximal phalanx and distal phalanx
Cerveri et al., 2008	Single markers	9	Hemispherical	- Forearm: Radial/ulnar styloid process and dorsal part of the forearm -Hand: MCP of 3rd metacarpal, MCP of 4th metacarapal and center of the hand -Thumb: three markers on the CMC, 1 marker on thelateral protuberance of the distal head of the metacarpal bone, 2 markers 20 mm away from the CMC on the metacarpal surface
Chang and Pollard, 2008	Single markers	6	Hemispherical	−3 markers on the proximal end of the first metacarpal on the radial side, the distal end of he first metacarpal of the radial side and the distal end of the first metacarpal on the ulnar side −3 markers on the proximal end of the third metacarpal, the distal end of the third metacarpal and the distal end of the second metacarpal
Coppola et al., 2018	Cluster Single markers	2 × 3 markers 11	No specification	Cluster - On the dorsal side over the 3rd metacarpal bone between the wrist and the MCP joint - Rigid body on the dorsal side of the right forearm two inches proximal to the right wrist Single markers - Lateral and medial epicondyles, lateral and medial styloids, MCP joints 2–4 - Thumb: On CMC, MCP, IP-joint and tip
Curran et al., 2019	Single markers	12 markers	5mm, spherical	-Thumb (5 markers): Base of metacarpal bone, metacarpal head, radial surface of metacarpal head, proximal phalanx head, distal thumb nail over distal phalanx head -Digit 2–5 (4 markers): Distal fingernail -Index finger (2 markers): Base of metacarpal, head of metacarpal -Middle finger (1 marker): Head of metacarpal
Gustafsson et al., 2018	Cluster Single markers	1 × 3 tracking markers 1 tracking marker, 9 calibration m	Hand: 9 mm Thumb: 5 mm Spherical	- Calibration markers: Ulnar/radial styloid, lateral and medial aspect of interphalangeal and metacarpal joints - Hand markers: One marker on third metacarpal bone, two markers placed mid-segment - Thumb: Marker cluster on proximal phalanx and one marker on the fingernail
Hamann et al., 2014a	Cluster (tripods)	3 × 3 markers (on pins)	No specification Spherical	- Thumb: Metacarpal bone and on proximal phalanx - Index finger: Metacarpal bone
Hamann et al., 2014b	Cluster (tripods)	3 × 3 markers (on pins)	No specification Spherical	-Thumb: Metacarpal bone and on proximal phalanx -Index finger: Metacarpal bone
Kodama et al., 2021	Cluster Single marker	3 × 3 markers (on T-shaped plate) 1	No specification Spherical	-Thumb: nail marker -T-shaped cluster on proximal phalanx and metacarpal bone of the thumb -T-shaped cluster on the third metacarpal bone -Marker attachment from Lin et al., 2011 [20]
Li and Tang, 2007	Single markers	6	5 mm Spherical	- Thumb: Proximal metacarpal bone, MCP joint, IP joint, fingertip - thumb: t-shaped plates (with 1 marker each) on metacarpal and proximal phalanx
Lin et al., 2011	Cluster Single markers	4 × 3 markers (on T-shaped plate) 2	4 mm Spherical	Cluster -On the proximal phalanx of the thumb, on the first metacarpal bone and on the styloid process of the radial bone (=trapezium; but only attached when in static, neutral position) - On the third metacarpal bone Single markers - Thumb: Fingertip and 1 cm distal to IP joint
Marquardt et al., 2014	Cluster (tripods) Single markers	2 × 3 markers (on pin and T-shaped plate) 4	No specification Spherical	Cluster - On the thumb nail - On the second metacarpal bone (T-shaped plate) Single markers - two markers on the proximal phalanx (thumb) - two markers on the first metacarpal bone - Marker placement as suggested by Metcalf et al., 2008
Tang et al., 2008	Single markers	6	5 mm Spherical	- Marker attachment following Li and Tang, 2007 - Thumb: Proximal part of the first metacarpal, MCP joint, IP joint, fingertip - Thumb: T-shaped plates with one marker on the metacarpal bone and the proximal phalanx
Zhang et al., 2005	Single markers	21	5 mm Spherical	- Thumb: CMC, MCP, IP joints and fingertip - Digit 2–5: MCP, PIP, DIP joint and fingertip - One marker on CMC 3
Long finger models				
Degeorges et al., 2005	Cluster (tripod) Single markers	3 × 3 markers (on pins) 3	10 mm 17 mm Spherical	- One tripod per proximal, middle and distal phalanx on a digital cast - Single markers on the fist cast
Gharib et al., 2015	Single markers	Not clearly stated, probably 19	12 mm, spherical	- For finger abduction: 1 marker over DIP of the measured finger, 2 markers on DIP and MCP of reference finger (reference finger = finger next to measured finger)
Hensler et al., 2020	Cluster	2 × 4 markers (on rigid plate)	3 mm Hemispherical	- Proximal and middle phalanx (Weight via a cable-pulley-system on the DIP joint)
Lee and Jung, 2015	Single markers (1–3 per segment) Cluster	(1) 24 (2) 38 (3) 48 (4) 48, 3 markers per cluster	4 mm, hemispherical	-Four types of marker attachment methods (1)One marker per joint (24 markers): On heads of MCP, IP, PIP, DIP and fingertip, proximal heads of CMC 2,3,4,5 (2)two markers per segment (38 markers): Distal and proximal segment of each segment, proximal heads of CMC 2,3,4,5 (3)three markers per segment (triangular shape): Triangular shape on each segment (4)Cluster markers and T-shaped plates
Lester et al., 2012	Single markers	34	Wrist: 8.5 mm, hand: 5 mm, phalanx: 3 mm Hemispherical	−4 markers on the wrist −6 markers for the hand - each phalanx was defined by two markers
Metcalf and Notley, 2011	Single markers Virtual marker	26 1	3 mm, hemispherical	-Placed on forearm, wrist -First, second and fifth carpometacarpal joints -One marker on each MCP, PIP, DIP -Markers on the fingertips
Metcalf et al., 2020	Single markers Virtual marker	26 1	3 mm Hemispherical	- Wrist: Distal radius/ulna, radial/ulnar styloid - CMC 1, 2 and 5 - MCP joints, PIP joints, DIP joints and fingertips
Speirs et al., 2001	Single markers	5	n.s	−2 markers on third metacarpal bone −1 marker on second metacarpal bone −2 markers on proximal phalanx of the index finger
Wang et al., 2013	Single markers	13	9 mm Spherical	- Index: Fingertip, DIP, PIP, MCP, midpoint of metacarpal bone - Middle finger: Fingertip, DIP, PIP, MCP, midpoint of metacarpal bone - Wrist: Ulnar/radial styloid process, dorsal wrist
Warlow and Lawson, 2012	Single marker Virtual markers	12 8	4 mm Hemispherical	- Index finger: 3 markers on each phalanx segment −3 markers on the dorsal surface of the hand - Medial and lateral point of the finger joints to define joint centers

The clusters typically consisted of three markers, with four exceptions (Leitkam et al., 2014; Leitkam and Reid Bush, 2015; Coupier et al., 2016; Hensler et al., 2020), where the clusters had four markers. Additionally, six studies mentioned the use of virtual markers (Metcalf et al., 2008; Metcalf and Notley, 2011; Cocchiarella et al., 2016; Lee and Jung, 2016; Metcalf et al., 2020). In all cluster studies, one cluster per segment was applied onto the center of the segments, while two studies placed the clusters on the dorsal side directly onto the joints (Hamann et al., 2014a; Hamann et al., 2014b). In three studies the clusters were additionally placed on pins (Degeorges et al., 2005; Marquardt et al., 2014; Coupier et al., 2016).

Thirteen studies used a single marker on the fingertip. In 18 studies there was a single marker placed directly onto the dorsal side of the finger joints, while one study placed the markers close to the joints (Baker et al., 2007). Two studies described the use of an additional marker placed in the middle of the segment (Speirs et al., 2001; Lee and Jung, 2016), four studies described the use of two markers per segment (Lin et al., 2011; Lester et al., 2012; Nataraj and Li, 2013; Marquardt et al., 2014) and in four studies, three single markers were placed on one segment for the index (Warlow and Lawson, 2012) and the CMC of the thumb (Cerveri et al., 2008; Chang and Pollard, 2008; Curran et al., 2019). Lee et al. (Lee and Jung, 2015) compared one, two and three markers per segment, which were placed onto the segments of the long fingers. Table 5 shows a detailed description of the marker setup.

Some included studies refer to each other according to the applied marker set. Tang et al. (2008) refer to the work of Li and Tang (2007) while Kodama et al. (2021) refer to Lin et al. (2011) for their utilized marker set. Moreover, Marquardt et al. (2014) applied a marker arrangement proposed by Nataraj and Li (2013), while Baker et al. (2007) refer to Cook and colleagues (2007). Additionally, the studies of Hamann et al. (2014a); Hamann et al., 2014b and Leitkam et al. (2014); Leitkam and Reid Bush, 2015) use the same marker set approach, respectively. Last but not least, the work of Metcalf and Notley (2011); Metcalf et al., 2020 is a development of an earlier work from the same research group (Metcalf et al., 2008).

### 3.7 DoF, coordinate system, kinematic approach, validation and reliability

In most studies, there was no specific information about the total number of DoF, however, DoF could be assessed due to the information of the number of segments and joint definitions (Table 6). Three studies modeled all joints of the thumb (CMC, MCP, IP) as a three DoF joint (Goislard de Monsabert et al., 2014; Sancho-Bru et al., 2014; Cocchiarella et al., 2016), while some modeled either the CMC or/and the MCP as 3 DoF. Another combination that occurred frequently was modeling the CMC, MCP and IP as three, two and one DoF, respectively (Nataraj and Li, 2013; Marquardt et al., 2014; Lee and Jung, 2016; Ma’touq et al., 2018; Kodama et al., 2021). In two studies, the IP joint of the thumb was modelled as a two DoF joint (Zhang et al., 2005; Ma’touq et al., 2018). Six studies modelled the MCP of the thumb as two DoF (Zhang et al., 2005; Baker et al., 2007; Cerveri et al., 2007; Cook et al., 2007; Cordella et al., 2017; Ma’touq et al., 2018). Coupier et al. (2016) defined a “thumb basis joint”, including three DoF, which represent both the CMC and MCP of the thumb. For the long fingers, five models defined all joints (MCP, DIP, PIP) as three DoF (Degeorges et al., 2005; Goislard de Monsabert et al., 2014; Sancho-Bru et al., 2014; Lee and Jung, 2015; Cocchiarella et al., 2016), while Lee and Jung (2016) modelled only the MCP as a three DoF joint. The DIP and PIP were defined as a 2 DoF joint in one study (Metcalf and Notley, 2011). More details of the DoF description are shown in Table 6.

**TABLE 6:** Model description (quantity of segments, DOF, coordinate system and rotation matrix, ISB guidelines).

First author	Quantity of segments	DoF per joint	Total DOF	Coordinate system and rotation matrix	Kinematic approach	Guidelines
Baker et al., 2007	11	Digit 2–5 MCP 2 (flexion/extension and abduction/adduction) PIP 1 (flexion/extension) Thumb MCP 2 (flexion/extension and abduction/adduction) IP 1 (flexion/extension)	15	Local palm reference frame based on 3 hand markers, while 5 vectors representing proximal phalanges, which are projected onto the hand’s local sagittal and coronal plane No defined rotation matrix	Vector calculation	---
Cerveri et al., 2007	17 (incl. Forearm)	Digit 2–5 MCP: 2 (flexion/extension and abduction/adduction), PIP/DIP: 1 (flexion/extension) Thumb: CMC 2 (flexion/extension and abduction/adduction), MCP 2 (flexion/extension and abduction/adduction), IP 1 (flexion/extension) Wrist: 2 (flexion/extension, abduction/adduction)	23	Anatomical calibration frame wrist: Y: Pronation/supination, Z: “Palm plane normal direction”, X: “Right hand rule” Digit 2–5: X: “Normal direction to the plane defined by the 4 markers on MCP, PIP, DIP, and FT of the corresponding finger”, Y, Z not clear thumb: X: “Normal direction to the plane defined by the 4 markers at TM, MCP, IP and FT”, Z: “The intrinsic pronation respect to the palm, is obtained thanks to an additional marker, positioned on a rigid structure bound to the metacarpal segment and directed out-ward from a flat open palm”	Forward kinematics approach	---
Cocchiarella et al., 2016	16, 18, 20 (incl. Forearm)	Digit 2–5 MCP 3 (pronation/supination, abduction/adduction, flexion/extension) Thumb CMC, MCP, IP 3 (flexion/extension, abduction/adduction, pronation/supination)	21	Anatomical calibration frame Euler angles - cardan sequence (X-Y-Z: flexion/extension – abduction/adduction – pronation/supination)	Direct kinematics	Refer to ISB guidelines, but use a different axis definition
Cook et al., 2007	7 (Incl. Forearm)	Thumb: MCP 2 (flexion/extension, abduction/adduction) Digit 2–5: MCP 2 (flexion/extension, abduction/adduction), PIP 1 (flexion/extension) Wrist: 2 (ulnar/radial deviation, flexion/extension)	17	Anatomical calibration frame -Origin: Midpoint between handmarker 1 and 2 -Z: from handmarker 2 to handmarker 1 (flexion/extension) -Y: perpendicular to the plane of the handmarkers (abduction/adduction) -X: cross product of Y- and Z-axes (pronation/supination)	Vector analysis	---
Cordella et al., 2017	16	Thumb: CMC, MCP 2 (flexion/extension, abduction/adduction) IP1 (flexion/extension) Digit 2–5: MCP 2 (flexion/extension, abduction/adduction), PIP/DIP 1 (flexion/extension)	21	Anatomical calibration frame Z: flexion/extension Y: abduction/adduction X: pronation/supination Euler angles Z-Y-X	Forward kinematics approach	---
Coupier et al., 2016	20 (Incl. Forearm and metacarpal II-V)	Digit 2–5 MCP 3 (flexion/extension, abduction/adduction, int/ext rotations) PIP/DIP 3 (flexion/extension, abduction/adduction, int/ext rotations) Thumb TBJ (thumb basis joint) 3 (abduction/adduction, flexion/extension, int/ext rotation), MCP, IP 3	45	Anatomical calibration frame X: abduction/adduction Y: internal/external rotation Z: flexion/extension Euler angles - cardan sequences (Z-Y-X sequence and X-Z-Y sequence for flexion/extension and adduction/abduction movements, respectively)	Direct kinematics	ISB guidelines
Goislard de Monsabert et al., 2014	17 (incl. Forearm)	Digit 2–5 MCP, PIP, DIP 3 (flexion/extension, abduction/adduction, pronation/supination) Thumb CMC, MCP, IP 3 (flexion/extension, abduction/adduction, pronation/supination)	45	Comparison of different local frame definitions (“reference”, “landmark”, “functional”) X: transverse axis, radially oriented Y: longitudinal axis, proximally oriented Z: sagittal axis, dorsally oriented Euler angles - cardan sequence (X-Z-Y: flexion/extension – abduction/adduction – pronation/supination)	Direct kinematics	Critical review of ISB guidelines and alternative proposal
Lee and Jung, 2016	16	Digit 2–5: MCP 3 (flexion/extension, abduction/adduction, axial rotation), PIP, DIP 1 (flexion/extension) Thumb: CMC 3 (flexion/extension, abduction/adduction, axial rotation), MCP 2 (flexion/extension, abduction/adduction), IP 1 (flexion/extension)	26	Anatomical calibration frame Z: Flexion/extension Y: Abduction/adduction X: Axial rotation Euler angles - cardan sequence (Z-Y-X)	Direct kinematics	Refer to ISB guidelines, but use a different axis definition
Leitkam et al., 2014	16	Digit 2–5: MCP 2 (flexion/extension, abduction/adduction), PIP/DIP 1 (flexion/extension) Thumb: CMC 2 (flexion/extension, abduction/adduction), MCP 1 (flexion/extension), IP 1 (flexion/extension)	20	Anatomical calibration frame Z: flexion/extension X: abduction/adduction Y: supination/pronation Euler angles Z-X-Y For the theoretical model a denavit-hartenberg transformation was used	Direct kinematics	ISB guidelines
Leitkam and Reid Bush, 2015	16	Digit 2–5: MCP 2 (flexion/extension, abduction/adduction), PIP/DIP 1 (flexion/extension) Thumb: CMC 2 (flexion/extension, abduction/adduction), MCP 1 (flexion/extension), IP 1 (flexion/extension)	20	Anatomical calibration frame Z: flexion/extension X: abduction/adduction Y: supination/pronation Euler angles Z-X-Y For the theoretical model a denavit-hartenberg transformation was used	Direct kinematics	ISB guidelines
Ma’touq et al., 2018	19	Thumb CMC 3 (flexion/extension, abduction/adduction, axial rotation) MCP, IP 2 (flexion/extension, abduction/adduction) (ab/ad is passive for IP) Digit 2–5 MCP 2 (flexion/extension, abduction/adduction), PIP, DIP 1 (flexion/extension)	26 Incl. 4 of the palm arc	Anatomical calibration frame X: proximal-distal axis Y: radial-ulnar axis Z: palmar-dorsal axis Denavit–Hartenberg convention	Direct as well as forward and inverse kinematics	---
Metcalf et al., 2008	19 Incl. Forearm	Digit 2–5: MCP 2 (flexion/extension, abduction/adduction), PIP (flexion/extension), DIP (flexion/extension) Thumb: MCP/IP 1 (flexion/extension), CMC 2 (abduction/adduction and rotation) Wrist: 2 (flexion/extension, radial/ulnar deviation)	23 Incl. 2 at the wrist	Anatomical calibration frame Wrist X: flexion/extension Y: pronation/supination Z: abduction/adduction Digits X: pronation/supination Y: flexion/extension Z: abduction/adduction	Vector analysis	Critical review of ISB guidelines and alternative proposal
Nataraj and Li, 2013	8	Thumb CMC 3 (flexion/extension, abduction/adduction, axial rotation) MCP 2 (flexion/extension, abduction/adduction) IP 1 (flexion/extension) Index MCP 2 (flexion/extension, abduction/adduction) PIP, DIP 1 (flexion/extension)	10	Functional calibration frames X: oriented to right (ulnar) Y: oriented dorsally Z: oriented proximally Euler angles – Cardan sequence (X-Y-Z)	Inverse kinematics	Refer to ISB guidelines, but use a different axis definition
Sancho-Bru et al., 2014	Not clear	Digit 2–5: MCP, PIP, DIP Digit 4 and 5: CMC Thumb: CMC, MCP, IP 3 DOF per joint (flexion/extension, abduction/adduction, pronation/supination)	54 incl.3 at the wrist	Anatomical calibration frame Z: flexion/extension X: abduction/adduction Y: pronation/supination Euler angles - cardan sequence (Z-X-Y)	Direct kinematics	ISB guidelines
Thumb models						
Carpinella et al., 2006	12	Digit 2–5: MCP, PIP 1 (flexion/extension) -Thumb: MCP 2 (anteposition and abduction), IP 1 (flexion angle)	11	Anatomical calibration frame X: flexion/extension Y: pronation/supination Z: abduction/adduction	Vector analysis	---
Cerveri et al., 2008	2	Thumb: CMC 2 (flexion/extension, abduction/adduction)	2	Anatomical calibration frame -wrist: X: Flexion/extension, Y-axis and Z-axis were derived from the X-axis, by a graham–Schmidt orthogonalization to the markers points on the wrist -Hand dorsum: Z: Direction of the plane fitting the three markers on the hand, X and Y graham-schmidt orthogonalization -Thumb universal joint (UJ model): Y: Internal/external rotation (approximating F-E), X: Line joining markers M7 and M8 (approximating A-A), Z: Graham-schmidt orthogonalization of Y and X NN model (thumb) -Functional calibration frame -“two nonorthogonal and non-intersecting rotation axes, accounting for the F-E and the A-A, respectively” -Denavit–Hartenberg convention	Inverse kinematics	---
Chang and Pollard, 2008	2	Thumb: CMC 2 (flexion/extension, abduction/adduction)	2	Anatomical calibaration frame Sequencial rotations Flexion/extension of hand dorsum z-axis Pronation/Supination axis Adduction/Abduction of the thumb z-axis Custom transformation matrix; measured CMC joint configuration is related to the functional joint configuration by the two change-of-coordinate transforms; denavit-hartenberg convention	Optimisation approach Anatomical bases cost metric	---
Coppola et al., 2018	5	Thumb CMC 3 (abduction/adduction, flexion/extension, pronation/supination), MCP 2 (abduction/adduction, flexion/extension) IP 1 (flexion/extension)	6	Local reference frame using bony landmarks (“palpated, digitized, and tracked”) Euler angles (no specification)	Direct kinematics	No information in the paper
Curran et al., 2019	4	Thumb: CMC 2 (flexion/extension, abduction/adduction), MCP/IP 1 (flexion/extension)	4	Local anatomical defined joint coordination systems X: pointing radial Z: pointing distal y: Orthogonal to the plane of the hand	Direct kinematics	---
Gustafsson et al., 2018	2	Thumb MCP 3 (flexion/extension, abduction/adduction, rotation)	3	Anatomical calibration frame Z: axis between medial and lateral MCP joint markers Y: axis between midpoint of MCP joint markers and midpoint of IP joint markers X: cross product of z- and y-axes Euler angles - cardan sequence (Z-X-Y: flexion/extension – abduction/adduction, rotation)	Direct kinematics using a customized model written in the software body builder (vicon)	ISB guidelines
Hamann et al., 2014a	3	Thumb CMC, MCP 3 (flexion/extension, abduction/adduction, pronation/supination)	6	Anatomical calibration frame X: flexion/extension Y: abduction/adduction Z: Pronation/supination Euler angles - cardan sequence (X-Y-Z)	Direct kinematics	---
Hamann et al., 2014b	3	Thumb CMC, MCP 3 (flexion/extension, abduction/adduction, pronation/supination = axial rotation)	6	Anatomical calibration frame X: flexion/extension Y: abduction/adduction Z: Pronation/supination Euler angles - cardan sequences (X-Y-Z)	Direct kinematics	---
Kodama et al., 2021	4	Thumb CMC 3 (flexion/extension, abduction/adduction, supination/pronation) MCP 2 (flexion/extension, abduction/adduction) IP 1 (flexion/extension)	6	Anatomical calibration frame Coordination system definition used ISB conventions Euler angles – Cardan sequence (X-Y-Z)	Direct kinematics	ISB guidelines
Li and Tang, 2007	4	Thumb CMC 3 (flexion/extension, abduction/adduction, pronation/supination), MCP 3 (flexion/extension, abduction/adduction, pronation/supination) IP 1 (flexion/extension)	7	Anatomical calibration frame X: Flexion/extension Y: Abduction/adduction Z: Pronation/supination Euler angles - cardan sequences (X-Y-Z)	Direct kinematics	---
Lin et al., 2011	5	Thumb CMC 3 (flexion/extension, abduction/adduction, axial rotation) MCP 3 (flexion/extension, abduction/adduction, axial rotation) IP 1 (flexion/extension)	7	Anatomical calibration frame Z: Flexion/extension X: Abduction/adduction Y: internal/external rotation Euler angles - cardan sequence (X-Z-Y)	Vector analysis to calculate the IP joint angle Otherwise direct kinematics	ISB guidelines
Marquardt et al., 2014	5	Thumb CMC 3 (flexion/extension, abduction/adduction, supination/pronation), MCP 2 (flexion/extension, abduction/adduction) IP 1 (flexion/extension)	6	Functional calibration frame for IP, MCP, CMC X: ulnar/radial axis → flexion/extension Y: dorsal/palmar axis → abduction/adduction Z: proximal/distal axis → supination/pronation Euler angles - cardan sequence (X-Y-Z)	Direct kinematics	Refer to ISB guidelines, but use a different axis definition
Tang et al., 2008	4	Thumb CMC 3 (flexion/extension, abduction/adduction, pronation/supination), MCP 3 (flexion/extension, abduction/adduction, pronation/supination) IP 1 (flexion/extension)	7	Anatomical calibration frame X: flexion/extension Y: abduction/adduction Z: pronation/supination Euler angles - cardan sequences (X-Y-Z)	Direct kinematics	---
Zhang et al., 2005	16	Thumb CMC 3 MCP, IP 2	7	Anatomical calibration frame Local coordinate system (X-Y-Z) X: projection of CMC3 – MCP3 vector Y: axis pointing radially Z: pointing dorsally No rotation matrix mentioned	Direct kinematics and globographic representation method for MCP, IP and DP markers (spheres least square fitting)	---
Long finger models						
Degeorgeset al., 2005	13	Digit 2–5 MCP, PIP, DIP 3 (flexion/extension, abduction/adduction, longitudinal axial rotations = supination/pronation)	36	Anatomical calibration frame Y: flexion/extension Z: abduction/adduction X: longitudinal axial rotation “Mobile axis method”, refers probably to classical euler angles with cardan sequence Y-Z-X	Direct kinematics	---
Gharib et al., 2015	10	-Digit 2–4: MCP 2 (abduction/adduction, flexion/extension), PIP 1 (flexion/extension) -Thumb: CMC 1 (abuction/adduction)	13 (incl. 1 at CMC 1)	No information	No information	---
Hensler et al., 2020	2	Index and middle finger: PIP 1 (lateral deviation)	2	Anatomical calibration frame Local reference frame for each marker set, orientation according to ISB guidelines No rotation matrix mentioned	Direct kinematics	ISB guidelines
Lee and Jung, 2015	16	Digit 2–5: MCP 2 (flexion/extension, abduction/adduction), PIP/DIP 1 (flexion/extension)	12	X: pronation/supination Y: abduction/adduction Z: flexion/extension Euler angles (X-Y-Z) (for three markers per segment and cluster marker methods), cheng and Pearcy’s equation for one and two markers per segment	Direct kinematics	ISB guidelines
Lester et al., 2012	14 (incl. Forearm)	Digit 2–5 MCP 2 (flexion/extension, abduction/adduction)	8	No specification	Direct kinematics	No specification
Metcalf and Notley, 2011	19 (incl. Forearm)	Digit 2–5: MCP/PIP/DIP 2 (flexion/extension, abduction/adduction)	23 Incl. 2 at the wrist	Anatomical calibration frame -three planes for the hand: radial hand plane (RHP), middle hand plane (MHP, ulnar hand plane (UHP) -A plane is defined for each finder -Finger movement is then calculated relative to specific hand plane Index finger - > RHP 3rd and 4th finger - > MHP 5th finger - > UHP	Vector analysis	---
Metcalf et al., 2020	19 (incl. Forearm)	Digit 2–5 PIP, DIP 1 (flexion/extension)	8	Joint coordinate system based on CT scans (including marker positions) Y: palmar-dorsal axis X: proximal-distal axis Z: radial-ulnar axis Rodriguez rotation	Reference to the modified HAWK method (Metcalfet al., 2008) applying vector calculations	---
Speirs et al., 2001	2	MCP 2 (flexion/extension, abduction/adduction)	2	Anatomical calibration frame Coordinate system for hand segment (I, J, K) and the proximal phalanx (i, j, k) K, k: Pointing radially in a left hand, ulnarward in a right hand (flexion/extension) I, i: Pointing proximally (pronation/supination) J, j: Abduction/adduction Euler angles with extension/flexion - adduction/abduction - pronation/supination sequence “no rotation” assumption at the MCP joint (“locked” pro-/supination)	Direct kinematics and vector analysis	---
Wang et al., 2013	8	Index MCP 2 (flexion/extension, abduction/adduction), PIP, DIP 1 (flexion/extension) Middle MCP 2 (flexion/extension, abduction/adduction), PIP DIP 1 (flexion/extension)	10 Incl. 2 at the wrist	Anatomical calibration frame, no information on axis definition Euler angles - cardan sequences (X-Z-Y)	Flexion angle of the PIP and MP joint using vector calculation Otherwise direct kinematics	---
Warlow and Lawson, 2012	4	Index MCP 2 (flexion/extension, abduction/adduction) PIP, DIP 1 (flexion/extension)	4	Functional calibration frame Y: sagittal plane X: directed palmarly Z: flexion/extension No rotation matrix mentioned	Inverse kinematics (“transformation technique” to define axes of rotation)	ISB guidelines

All but three studies (Baker et al., 2007; Lester et al., 2012; Gharib et al., 2015) defined a coordinate system, while 10 of them followed the guidelines of the ISB. Four studies referred to the ISB guidelines but used a different axis definition (Nataraj and Li, 2013; Marquardt et al., 2014; Cocchiarella et al., 2016; Lee and Jung, 2016). Some studies used functional calibration movements to define the joint coordinate system (Warlow and Lawson, 2012; Nataraj and Li, 2013; Goislard de Monsabert et al., 2014; Marquardt et al., 2014), though, most studies used an anatomical frame approach.

While the ISB recommendations appear to be the only established guidelines for marker placement and coordinate frame definitions of the hand and finger segments published by a leading society, two studies raise strong arguments underscoring the need for continued refinement. Both Metcalf et al. (2008) and Goislard de Monsabert et al. (2014) emphasize, that some anatomical reference points required for accurate modeling are inaccessible to surface markers and difficult to estimate experimentally. The latter study compared three different local frame definitions with each other (reference frame, functional frame and landmark frame adapted from the ISB guidelines).

Twenty-five studies gave information about the applied rotation matrix, with Euler angles in Cardan sequences being the most frequently used (Table 6). Five studies used the Deanvit-Hartenburg convention (Cerveri et al., 2008; Chang and Pollard, 2008; Leitkam et al., 2014; Leitkam and Reid Bush, 2015; Ma’touq et al., 2018), while one used the Rodriguez rotation (Metcalf et al., 2020). Most studies applied a classical direct kinematics approach to calculate joint angles, however, some used vector calculations (Speirs et al., 2001; Carpinella et al., 2006; Baker et al., 2007; Cook et al., 2007; Metcalf et al., 2008; Lin et al., 2011; Metcalf and Notley, 2011; Wang et al., 2013; Metcalf et al., 2020) or an inverse kinematic fitting approach (Cerveri et al., 2008; Warlow and Lawson, 2012; Nataraj and Li, 2013; Ma’touq et al., 2018). Two studies used forward dynamics (Cerveri et al., 2007; Cordella et al., 2017), while one study used an “optimization approach” based on anatomical cost metrics (Chang and Pollard, 2008) (Table 6).

Ten studies did a validation of the hand model using goniometers, CT scans and estimated data (Cook et al., 2007; Leitkam et al., 2014; Sancho-Bru et al., 2014; Lee and Jung, 2015; Coupier et al., 2016; Cordella et al., 2017; Ma’touq et al., 2018; Curran et al., 2019; Metcalf et al., 2020), while Metcalf (Metcalf et al., 2008) conducted a validation only for the wrist. Eight studies conducted a reliability test (Speirs et al., 2001; Carpinella et al., 2006; Cerveri et al., 2007; Metcalf et al., 2008; Sancho-Bru et al., 2014; Coupier et al., 2016; Cordella et al., 2017; Hensler et al., 2020) (Table 3).

## 4 Discussion

This systematic review identified 38 studies, which included model definitions of the (partial) hand to analyze joint kinematics of the finger joints. Four models achieved the maximum quality rating in our assessment: one full-hand model (Lee and Jung, 2016), two thumb models (Gustafsson et al., 2018), and one long finger model (Carpinella et al., 2006; Lee and Jung, 2015), however, only one study validated its method (Lee and Jung, 2015) and tested its method on two patients after stroke (Carpinella et al., 2006), highlighting a lack of clinical verification.

In general, it could be shown that there are several approaches of marker-based movement analysis of the hand, which differ mainly in the arrangement of their markers as well as the number of DoF. Furthermore, the review aimed to potentially identify existing hand models that are suitable for the clinical practice. Interestingly, only one study of the included full hand models tested its marker protocol on a patient population (Leitkam and Reid Bush, 2015), while all others were tested on healthy groups. While thumb models including CMC were mainly applied to patients with osteoarthritis or carpal tunnel syndrome, models focusing solely on the long fingers were tested across a more diverse set of clinical populations, including individuals with rheumatoid arthritis, joint-specific osteoarthritis, finger injuries, and post-stroke impairments.

A general limitation is the lack of practically applicable guidelines for marker-based hand motion analysis. While some studies referred to the ISB recommendations as a useful initial reference, these guidelines were not consistently applied in model development. Several authors also highlighted that certain anatomical landmarks required by current standards cannot be reliably captured using external surface markers. Furthermore, depending on the intended application, a comprehensive hand model does not necessarily require all possible degrees of freedom. In some cases, simplifying joint representations may actually enhance feasibility and reproducibility without significantly compromising the validity of the analysis (Metcalf et al., 2008; Goislard de Monsabert et al., 2014).

In the following different aspects of the systematic review are examined in detail.

### 4.1 Validity and reliability

Validity and reliability of the models were only sporadically examined. Validity was assessed using goniometers, CT scans, and a comparison of estimated and measured data (Sancho-Bru et al., 2014; Coupier et al., 2016; Ma’touq et al., 2018; Metcalf et al., 2020). Mostly, static or simple movements were used for validation like static evaluation using a mock-up hand (Lee and Jung, 2015) or specific postures with different joint angels (Cook et al., 2007). Among the highest-rated studies, only one study explicitly investigated the validity of the proposed model. As a result, the robustness of the remaining models in terms of validity cannot be adequately assessed based on the current evidence base. This represents a significant limitation, as both validity and reliability are essential for determining the methodological quality and clinical applicability of biomechanical models.

Goniometer measurements are highly dependent on the examiner, and capturing joint angles in a 3D space throughout the entire movement with manual goniometers measuring in a 2D fashion is challenging (Reissner et al., 2019b). Given the small anatomy of the hand, accurately applying goniometers to the joints being measured is difficult, potentially restricting the participant’s movement and distorting the results (Somia et al., 1998; Sancho-Bru et al., 2014; Reissner et al., 2019b). As noted by Sancho-Bru et al. (2014), goniometers cannot measure all joint angles simultaneously, and even small-segment joints, such as the DIP, are beyond their capability. Validation through imaging methods seems more suitable, though, the small hand anatomy and overlapping bones of the palm and fingers might pose a challenge as well. Additionally, a disadvantage is the use of ionizing radiation, increasing the risk of bodily harm and therefore raising ethical concerns (Metcalf et al., 2020). Additionally, detecting dynamic movement is challenging in this approach (Ma’touq et al., 2018).

An accurate method for assessing joint angles also involves the invasive placement of pins into the skin to avoid errors such as skin displacement. However, this may be associated with pain and an increased risk of infection (Fuller et al., 1997). Furthermore, it is associated with ethical concerns, as the insertion of pins into the skin is an invasive method. In general, soft tissue artifacts represent a significant source of error in motion analysis involving skin markers. As highlighted by Kontaxis et al. (2009), addressing these artifacts is a critical step in developing a motion analysis protocol. If soft tissue artifacts are insufficiently addressed, it should be an issue in future applications.

Only three articles included reliability testing. Coupier et al. (2016) evaluated inter-rater reliability and intra-rater reliability by having multiple examiners apply the marker set at different time points. This provides valuable insights into whether the placement of markers varies between different users, though, Coupier et al. used cadaver hands to test reliability. Similar testing was undertaken by Sancho-Bru et al. (2014), who tested the reliability for a grasping and raising task. Their global repeatability RMSE error lay between 1.6 and 3.2 for within session and between 2.3° and 3.8° in-between sessions. In another study (Hensler et al., 2020), data were collected multiple times, resulting in intraclass correlation coefficients of >0.9. However, the tested movement was an isolated radial/ulnar stability of the PIP joint, while the assessments were made on the same day, which is not sufficiently independent for reliability testing. Instead, data collection on different days is recommended.

The lack of validity and reliability measures for various approaches, such as vector analysis vs. direct kinematics or different marker placement strategies, limits the ability to accurately compare the methods presented across the included studies. Future research is needed to systematically evaluate these approaches using standardized and robust validity and reliability measures.

### 4.2 Marker setup

When placing individual markers, the position onto the dorsal side of the joints or near this location is frequently used. This position is easy to locate, but the skin displacement is greatest at this point as shown in the validation study of Ryu et al. (2006). Marker sets with markers placed directly onto the joint appear susceptible to variability in placement and would require application at a standardized finger flexion angle. Among the studies receiving the highest methodological ratings, one study employed only single-marker placements, whereas the remaining studies used a combination of marker clusters and single markers. A noticeable trend was that studies analyzing a larger number of body segments generally utilized smaller markers. The use of smaller markers likely contributed to higher tracking accuracy by reducing marker overlap and occlusion, particularly in anatomically crowded regions and during complex movements. In contrast, studies focusing on a limited number of segments were able to use fewer and, in some cases, larger markers without substantially affecting measurement quality. This suggests that marker size and configuration should be selected according to the complexity of the movement task and the number of segments being analyzed, balancing measurement accuracy with practical considerations of marker placement and tracking.

In some studies, additional markers on T-shaped plates (Li and Tang, 2007; Tang et al., 2008) or markers on elevations (“floating markers”) (Lee and Jung, 2016) are applied centrally to each segment, which can improve the assignment of the segment and reduce the influence of the displacement of the skin but may also cause limitations in freedom of movement. In other studies that do not use markers on joints, two or three single markers are applied to a segment. This approach avoids increased skin displacement but results in an increased overall number of markers used, which might be problematic due to increased preparation time and the limited space on the finger surface. Using placement guidelines like the ISB recommendations of Wu et al. (2005) can minimize marker placement errors; however, even following such definitions might result in challenging marker placement, as the recommendations might be difficult to apply in some cases (e.g., anatomical landmarks defined for the metacarpals (Goislard de Monsabert et al., 2014)). Therefore, it is essential to precisely define the positions onto the hand where markers need to be placed, while keeping each limitation in mind for interpretation.

Other studies exclusively employ clusters in their marker sets. It has been shown that cluster, containing multiple markers, enhance the ability to capture rotations and translations (Duffell et al., 2014). Such clusters consist of three or four markers per cluster, while in some instances, the markers were additionally affixed to pins or rods, orienting them either upward or outward-upward (Hamann et al., 2014a; Hamann et al., 2014b; Nataraj and Li, 2015; Coupier et al., 2016). This approach aims to alleviate the challenge posed by a high marker count in confined spaces and reduce skin displacement through the broad bases of the clusters. However, the heightened complexity of the measurement setup, increased cluster weight, and the associated risk of detachment, along with potential subject mobility restriction, pose challenges. For instance, wands or plates may restrict mobility, potentially impeding complex movements required for activities of daily living.

In general, the use of clusters offers the advantage that markers that are obscured during measurement can be reconstructed more effectively afterwards. Moreover, a cluster can independently represent movements in three-dimensional space without relying on connections to other markers. Nevertheless, this method also confronts the issue of a high marker count per unit area and the associated increased risk of marker detachment. To address the respective limitations of each method, some studies combined cluster-based marker placement with individually placed markers. Choosing the marker placement, thus, highly depends on the subjects being measured, particularly concerning the size and flexibility of their hands. In a comprehensive study, Lee and Jung (2015) compared four marker models ranging from one to three individual markers to a T-shaped plate with four markers per segment. They concluded that while one marker per segment suffices for static analysis (no significant differences between models), at least three markers are advisable for dynamic assessments, when feasible.

### 4.3 Segmentation and degrees of freedom

The metacarpal bones of all four long fingers are typically consolidated into a single segment, deviating from the physiological arch of the palm, though this discrepancy holds in most cases little clinical relevance (Cerveri et al., 2007). However, some surgical treatments addressing hand pathologies can change the physiological arch of the palm, which can also lead to pain and functional impairments (Durdzińska Timóteo et al., 2023). In these cases, it would be beneficial to consider those changes when choosing the model or discussing the limitations. Only a few studies included single CMC joints of the long fingers (Sancho-Bru et al., 2014) or modelled the palm of the hand with more than one segment (Metcalf et al., 2008). This likely results from the difficulty of capturing proximal metacarpal landmarks with experimental markers (Goislard de Monsabert et al., 2014).

The first metacarpal bone is often treated as a distinct segment, given its anatomical differences and associated range of motion, especially due to the unique characteristics of the first CMC joint. The modeling of the first CMC joint remains a subject of ongoing debate due to its anatomical complexity. While often described as having three DoFs (Cerveri et al., 2007), only two are biomechanically independent, as axial rotation is coupled with flexion and abduction. In line with recent literature, this systematic review accepted models that included at least two DoFs at the first CMC joint to ensure functional aspects of thumb opposition were captured. Modeling the movement in the first CMC, therefore, is highly debated in the literature. Coupier et al. (2016) even highlighted that CMC displacement at the thumb cannot be explicitly analyzed using reflective markers, while it rather be a combined movement of all joints located at the basis of the thumb. Therefore, they decided to call the tracked movement “Thumb Basis Joint” rather than CMC.

The thumb is commonly modeled with two additional segments, exhibiting typical movements such as flexion/extension and abduction/adduction at the MCP joint, as well as flexion/extension at the IP joint. Some studies have explored axial rotation or lateral tilt in the IP joint as well (Degeorges et al., 2005; Sancho-Bru et al., 2014). Given that the physiological degrees of freedom at the MCP and IP joints of the thumb are limited to flexion and extension, and that these joints are typically stable in the frontal plane, abduction–adduction is not expected under normal conditions. A minimal degree of axial rotation may occur during flexion, but it is generally negligible. However, in pathological cases such as joint instability, non-physiological movements, particularly in the frontal plane, can become more pronounced, making the calculation of additional DoFs clinically relevant. Moreover, for functional tasks such as pinching and grasping, the orientation of the distal phalanx plays a critical role. Patients may compensate for restricted motion in one joint by increasing movement in adjacent joints. Therefore, 3D tracking of the distal thumb segment remains especially valuable for accurately assessing thumb function, both under physiological and pathological conditions.

The segmentation of the long fingers is straight forward as it usually involves three segments (MCP, DIP, PIP). In the MCP joint, the prevalent movements include flexion/extension and abduction/adduction. Occasionally, pronation/supination in the MCP joint has been investigated, although less frequently (Sancho-Bru et al., 2014; Cocchiarella et al., 2016). The PIP and DIP joints are typically represented with one DoF, enabling flexion/extension.

With regard to model complexity, the highest-rated studies generally employed detailed hand models consisting of up to 16 segments when multiple fingers were included in the analysis. Such models allow for a more comprehensive representation of hand kinematics and capture the complex interactions between individual digits. Consequently, these models also incorporated a greater number of degrees of freedom, enabling a more detailed assessment of movement patterns. In contrast, the two highest-rated studies that focused exclusively on the thumb utilized substantially fewer segments (five and two segments, respectively) and therefore included fewer degrees of freedom. This reduction in model complexity is likely attributable to the narrower scope of the investigated movement rather than methodological limitations. Since thumb-specific analyses require the representation of fewer anatomical structures, simpler models may be sufficient to accurately capture the relevant kinematics.

### 4.4 Coordinate system

In most studies, a local coordinate system has been defined to describe the orientation of segments in three-dimensional space during motion. Ten studies oriented themselves according to the ISB guidelines and four referred to the ISB guidelines but used a different axis definition. The ISB has been striving for standardization recommendations for presenting kinematic data since 1990 (Wu and Cavanagh, 1995). The primary objective is to enhance reproducibility and facilitate communication among researchers through standardization (Wu et al., 2005). For each bone in the hand, a unique coordinate system is established to accommodate defined movements along its axes. Using a rotation matrix, the new orientation of a segment in space is described.

Such guidelines are very useful, however, as already mentioned above, previous literature critically revised the ISB recommendations. Some of the segment coordinate system definitions are difficult to apply due to anatomical points, which are not easy to palpate or estimate. An example is the ISB definition of the metacarpal coordinate system, where the head and the base of the metacarpal are needed, though, are either too broadly defined or hidden in the palm tissue. Therefore, alternatives to the ISB recommendations can be clearly justified.

Euler angles were the most commonly employed method for constructing rotation matrices, almost always using Cardan sequences as a special case of Euler angles which are characterized by the use of three different axes of rotation (e.g., Y-X-Z or X-Z-Y not X-Y-Y). In general, Cardan angles have, therefore, the advantage that they allow a clearer distinction between the axes of rotation and are less susceptible to singularities. It has been shown (Goislard de Monsabert et al., 2014) that different local frame definitions (e.g., anatomical vs. functional), although resulting in orientation deviations to up to 24°, have only small differences (<7°) regarding the first Cardan angle, while the second and third Cardan angle showed much bigger deviations between different local frames. Therefore, it is crucial that the primary movement of a joint is represented by the first Cardan angle (i.e., the first axis to rotate).

### 4.5 Measurement equipment

Preferably, passive reflective markers were used (Goislard de Monsabert et al., 2014; Coppola et al., 2018) as they are smaller and lighter than active markers. This choice allows participants to experience less restriction in their movements, and the markers are less prone to detachment.

A notable trend observed across the included studies was the relationship between marker size and model complexity. Hand models representing the entire hand generally favored smaller markers, with a diameter of 4 mm being the most commonly reported size. This preference is likely related to the large number of markers required to define the numerous small joints and segments of the hand. In such models, smaller markers may help reduce marker overlap and occlusion while allowing accurate placement on anatomically constrained regions. In contrast, studies focusing exclusively on thumb kinematics tended to use slightly larger markers. As thumb models require fewer markers and involve a smaller measurement volume, the risk of marker interference is reduced. Larger markers may therefore be advantageous due to their improved visibility and detectability by the motion capture cameras. For models focusing on the long fingers, marker sizes varied considerably, ranging from 3 mm to 12 mm. Here, marker size appeared to depend not only on the anatomical region of interest but also on the total number of markers used. Larger markers were generally employed in studies with fewer overall markers and were often placed on less constrained anatomical locations such as the dorsum of the hand or the wrist. Overall, the findings suggest that marker size is commonly adapted to the complexity of the model and the available placement area, balancing tracking accuracy with practical considerations related to marker visibility and occlusion.

The markers must be always detectable by at least two cameras, a consideration that influences the selection of the number of cameras and their alignment. Most hand movements can be captured at frequencies between 60 and 100 Hz. Higher frequencies enable more precise recording of faster movements, although it comes with a more frequent occurrence of artifacts, which can be subsequently addressed through data filtering (Cerveri et al., 2007). Too low measurement frequencies may result in the possible omission of crucial data, leading to measurement inaccuracies.

Across the highest-rated studies, a consistent methodological pattern was observed. All studies employed passive marker-based motion capture systems, predominantly using established setups. These systems were combined with relatively high camera counts (ranging from 10 to 18 cameras), likely enhancing spatial reconstruction accuracy and reducing marker occlusion during complex movements. Sampling frequencies ranged between 50 and 250 Hz, reflecting adaptation to the expected movement dynamics and ensuring sufficient temporal resolution for biomechanical analysis.

### 4.6 Clinical application

Primarily, the selected studies measured healthy participants without pre-existing hand conditions, only one study, which applied a full hand model, included a patient group next to a healthy cohort. The clinical applicability of the investigated models is, therefore, currently limited by the small number of studies involving clinical populations. Of the 38 included studies, only seven included participants with specific pathological conditions, and three studies investigated individuals following an intervention. However, all of these studies employed cross-sectional designs and collected data at a single time point. In the intervention studies, no pre-intervention measurements were available, preventing the assessment of within-subject changes and treatment-related effects over time. While these studies provide initial evidence that movement analysis can differentiate between healthy and clinical populations, they offer limited insight into disease progression or intervention outcomes. Longitudinal studies with repeated measurements before and after treatment are needed to evaluate whether the proposed models are sensitive to clinically meaningful changes and suitable for monitoring patient outcomes in clinical practice.

In some cases of partial hand models, patients with pathologies such as rheumatoid arthritis, thumb carpometacarpal joint arthritis, finger joint arthritis, carpal tunnel syndrome, finger injuries or patients with post-stroke conditions were included. Especially considering arthritis and rheumatoid arthritis, motion analysis is feasible in less advanced stages of these conditions. However, these conditions can cause deformities of finger joints or significant joint swelling (Hensler et al., 2020; Phutane et al., 2021). Altered joints can complicate marker placement and palpation of anatomical reference points, leading to challenges in marker placement on the joints, which must be considered when selecting a model for certain patient groups (Lester et al., 2012) and might make the placement of a full hand model consisting of more than one marker per segment difficult. Using a reduced marker concept on patients with finger deformities (e.g., lateral deviations or hyperextensions), where the assumption is that markers are placed on a straight, central line on the fingers, however, might cause problems. In these cases, a vector-based approach was often applied and in those models with a one DoF joint, axial deviations make it impossible to distinguish between lateral deviation and flexion. This needs to be taken in consideration when choosing a model or interpreting the results.

Patients with spasticity represent another clinically relevant group frequently examined in movement analysis, albeit with a predominant focus on the lower limbs. In this context, kinematic data are commonly used to inform both therapy planning and spasticity treatment strategies (Jaspers et al., 2009). Spasticity of the upper extremity can also occur, particularly after a stroke (Kerkemeyer et al., 2017). The use of marker-based hand models in patients with spasticity, however, presents notable challenges, particularly in achieving accurate and stable marker placement. Pathological muscle activity and abnormal joint postures can compromise measurement precision and repeatability. However, studies such as those by Gharib et al. (2015) and Carpinella et al. (2006) demonstrate that meaningful hand kinematics can still be captured in post-stroke populations. While Gharib et al. showed functional improvements through therapeutic intervention, Carpinella et al. validated a repeatable kinematic protocol even under clinical conditions.

These findings highlight the potential for adapting standardized models to the specific needs of neurologically impaired patients; however, no model has been applied to analyze hand and finger movements in patients with spasticity. Since spasticity syndrome can be caused by various central nervous system impairments such as stroke, multiple sclerosis, or childhood damage (for example, infantile cerebral palsy), it is common. Given that impairments of the upper extremity significantly impact patients’ quality of life, further investigations are needed to evaluate hand models in this area.

Movements performed varied between simple actions such as repeated flexions/extensions or abductions/adductions and everyday movements like grasping different shapes and objects. Providing a detailed description of the performed movements’ aids in making studies reproducible. In some hand models, achieved joint angles were not the primary focus. Instead, other parameters were investigated, such as the frequency of a particular movement (Currà et al., 2004), maximum hand opening (Betti et al., 2018), or finger position while playing a musical instrument (Maidhof et al., 2013) or in relation to the rest of the body (Albrecht et al., 2014).

### 4.7 Limitations

A limitation of this review is that the literature search was restricted to a limited number of databases. Although the inclusion of additional databases may have identified further relevant studies and broadened the evidence base, database selection was constrained by institutional access rights. Given the clinical focus of the review, the selected databases were considered to provide adequate coverage of the literature relevant to the research question.

Given that the focus of this review was on the methodology of the studies, and not enough studies performed a statistical validity and reliability approach, conducting a meta-analysis or other statistical analysis was not feasible. Consequently, the evaluation and discussion were qualitative. The PRISMA guidelines could not be fully considered, as they mainly focus on the development of systematic reviews for intervention studies. To assess the quality of the studies, a customized assessment table with pre-defined criteria was created, adapted by the SWiM guidelines proposed in Campbell et al. (2020). These criteria were proposed, discussed, and established by three independent scientists (VE, MT, UT). The criteria are highly relevant for describing the studies, as the information they capture is significant for the quality insurance of the data as well as the reproducibility of the studies or hand models.

The focus on joint angles as an outcome parameter was chosen to provide an objective parameter that accurately represents the data and is the classic outcome parameter of 3D motion analysis. While joint angles may not always be the most informative variable, they are highly relevant in many cases. However, the current evidence does not allow conclusions about whether these models adequately balance accuracy and feasibility, or whether they can be considered suitable for clinical application in terms of the repeatability required to track clinical decline or improvement.

We applied minimum inclusion criteria regarding the degrees of freedom for specific joints to ensure clinical and anatomical relevance. While this may have led to the exclusion of some studies presenting full-hand models that did not meet these criteria, the intent was not to favor partial over full-hand models, but rather to prioritize functional accuracy. Accordingly, both full and partial hand models were included in this review. From a physiological standpoint, a well-constructed single-digit model that accurately represents joint mechanics can be more valuable than a full-hand model lacking anatomical detail. However, it should be acknowledged that models with fewer segments are subject to fewer technical constraints, such as the number of markers and cameras required.

## 5 Conclusion

Although a wide variety of hand motion models have been described in the literature, their clinical applicability remains difficult to assess, as only a limited number of studies have included patient populations and few models have undergone formal validity and reliability testing. For clinical applications, both a precise description of the protocol and evidence of reliability, repeatability, and accurate representation of finger joint mechanics are essential. This review provides clear criteria for reproducible reporting, offers a comprehensive comparison of existing approaches, and highlights important research gaps. A wide range of finger kinematic models is available in the literature, however, yet no single model stands out as universally applicable across clinical settings. Four models achieved the maximum quality rating in our assessment: one full-hand model, two thumb models, and one long finger model. However, only the thumb protocol by Carpinella et al. (2006) was tested in a clinical context, particularly on two post-stroke patients, while the other three high-rated models were evaluated exclusively in healthy participants. Additionally, only the study of Lee and Jung (2015) validated their model against a 3D printed hand. This gap underscores a major limitation in current research: models with reproducible methodological documentation rarely undergone validity and reliability testing and application in clinical populations. Given the complexity of finger and hand function and the diversity of clinical presentations, it is unlikely that a single model can meet all needs. Therefore, model selection should be guided by the target population, the clinical or research objective, and methodological trade-offs regarding marker setup, joint segmentation, and measurement accuracy. Further research is needed to validate existing models and test for reliability under clinical conditions and to develop adaptable frameworks. Improving methodological transparency and refining current standards, such as the ISB guidelines, will be essential to enable reproducibility and comparability across laboratories.

## Data Availability

The original contributions presented in the study are included in the article/supplementary material, further inquiries can be directed to the corresponding author.
